# Multifunctional Polyurethane Composites with Coffee Grounds and Wood Sawdust

**DOI:** 10.3390/ma16010278

**Published:** 2022-12-28

**Authors:** Przemysław Bartczak, Julia Stachowiak, Marta Szmitko, Aleksandra Grząbka-Zasadzińska, Sławomir Borysiak

**Affiliations:** Faculty of Chemical Technology, Institute of Chemical Technology and Engineering, Poznan University of Technology, Berdychowo 4, PL-60965 Poznan, Poland

**Keywords:** polymer composites, rigid polyurethane foam, coffee grounds, sawdust, mechanical and insulating properties

## Abstract

Currently, the fundamental activity that will allow for the development of an economy with closed circulation is the management of food waste and production waste for the preparation of biocomposites. The use of waste materials of natural origin allows for the creation of innovative composites with improved physicochemical and functional properties. The present investigation concerns the use of coffee grounds (2.5–20 wt.%) and oak sawdust (2.5–20 wt.%) as effective fillers of rigid polyurethane foam. Innovative composite materials, previously indebted in the literature, were subjected to the necessary analyses to determine the application abilities: processing times, free density, water absorption, dimensional stability, mechanical properties (compressive strength), thermal conductivity, morphology, and flame resistance. The results with respect to the mechanical tests turned out to be the key. Increasing the number of coffee additives has a positive effect on the compressive strength. The addition of this filler in the range of 5–15 wt.% increased the compressive strength of the composites, 136–139 kPa, compared to the reference sample, 127 kPa. The key parameter analysed was thermal conductivity. The results obtained were in range of the requirements, that is, 0.022–0.024 W/m·K for all used amounts of fillers 2.5–20 wt.%. This is extremely important since these materials are used for insulation purposes. The results of the burning-behaviour test have confirmed that the addition of renewable materials does not negatively affect the fire resistance of the received foams; the results were obtained analogously to those obtained from the reference sample without the addition of fillers. The height of the flame did not exceed 17 cm, while the flame decay time was 17 s for the reference sample and the composite with coffee grounds and 18 s for the composite with oak sawdust. In this work, the practical application of bioorganic waste as an innovative filler for the insulation of flooded polyurethane foam is described for the first time. The introduction of fillers of natural origin into the polymer matrix is a promising method to improve the physicochemical and functional properties of rigid polyurethane foams. Composites modified with coffee grounds and sawdust are interesting from a technological, ecological, and economic point of view, significantly increasing the range of use of foam in various industries.

## 1. Introduction

Polyurethanes (PUs) are among the most widely used groups of polymeric materials in industry. For this reason, the polyurethane market has undergone dynamic developments in recent years. The diverse spectrum of applications of polyurethane is due to the variety of raw materials from which they are obtained. These substances include polyols and isocyanates, along with additives such as catalysts, blowing agents, flame retardants, fillers, and others. When components and mutual volume ratios are varied, porous and composite materials, as well as elastomers, adhesives, and coatings, can be obtained. The most important commercial applications are polyurethane foams, which represent 67% of the total polyurethanes consumption. There are flexible, viscoelastic, and rigid foams, which account for 23% of all polyurethane production. They are characterized by good application properties [1,2,3,4]. Therefore, they have been used in many industries, such as furniture, automotive, or the production of insulation materials. This work focusses on the insulation of rigid polyurethane foams [2,3,4,5,6].

In recent years, the increasing use of polyurethane materials, their production, and the resulting waste have led to a tightening of environmental requirements. Therefore, polyurethane producers are looking for solutions favourable from an economic and ecological point of view. As a result, additives obtained from renewable sources are attracting more and more attention. A promising aspect that may reduce production costs and improve mechanical and physical properties is the incorporation of natural waste into polyurethane foams. This concept is also good for the environment and industry. It allows for the management of residual waste and the reduction of its constantly growing amount. Examples of this type of filler are coffee grounds and sawdust [5,6,7,8,9,10,11].

From a legendary discovery in 575 AD in Abyssinia (today’s Ethiopia), coffee has become one of the most valuable agricultural commodities traded on the world markets. The coffee tree is a tropical evergreen plant, grown in the so-called coffee belt that stretches between the Tropic of Cancer and the Tropic of Capricorn in Africa, Asia, and Central and South America. Arable coffee land is more than 10 million acres, and each bush is harvested three times per year. The main producers are Brazil, Vietnam, and Columbia. More than 60 varieties of coffee trees are grown around the world. The main species include the following: *Coffea arabica*, *Coffea liberica*, and *Coffea robusta* [12,13,14,15,16]. Both the processing of coffee fruits in producing countries and the processing of coffee beans are a source of unavoidable waste. The coffee industry is responsible for generating large amounts of residues, with coffee grounds (CG) being the most abundant. CG is the residual material obtained when ground beans are processed to obtain a coffee drink. Almost half of the world’s coffee production is processed to prepare ground coffee, which generates around 6 million tons of CG per year. In total, 650 kg of CG is estimated to be produced for each ton of coffee beans and 2 kg of wet CG is produced for each kg of ground coffee, and coffee shops and producers of ground coffee account for half of the production [17,18,19]. CG have found application as a fertilizer in soil composting and enrichment. They were used, among others, in the cultivation of edible mushrooms such as the oyster mushroom (*Pleurotus ostreatus*). Due to their composition, coffee grounds can also be used in the production of biofuels [20]. Several different processes for biodiesel production with the use of CG have been presented in the literature, including one-step, two-step transesterification and CG in situ transesterification [21]. Juliana Farinassi Mendes et al. [22] investigated the physicochemical properties of pectin films (HDM) enriched with the coffee grounds used. To develop biodegradable films and to use unused resources, the authors modified HDM with CG (5–20 wt.%). HDM/CG films were produced by continuous casting using a coating equipment. An in-depth physicochemical analysis of the obtained composites was performed. Morphology, thermal stability, barrier and mechanical properties as well as characteristic functional groups in the structure of the obtained materials were defined. The CG showed good dispersibility and good integration into the polymer matrix. The addition of 20% CG caused significant changes in the properties of the pectin-based film, allowing for an increase in heat stability and colour. The inclusion of CG significantly improved the water vapour transmission coefficient, improving or at least maintaining the physicochemical properties. Henri Vahabi et al. [23] conducted studies on the potential of coffee grounds as a flame retardant for polymeric materials. Their work is the first report on the use of coffee biowaste as a permanent flame retardant for epoxy resins, which are typically highly flammable polymers. CG was used in the research, including CG chemically modified with dimethyl phosphite (P-CG). To prepare the composites, the authors mixed predetermined amounts of ground coffee biowaste and epoxy resin and then added the appropriate hardener. After dosing the ingredients, the system was mixed and then poured into suitable moulds placed in an oven for 3 h at 70 °C to cure the composites. In the analysis using the flow calorimeter method of pyrolysis combustion, a significant reduction in the amount of heat released (by 40%) was observed. Furthermore, combustion tests showed the self-extinguishing nature of this sample, which confirmed the usefulness of coffee biowaste used as a polymer flame retardant. Meanwhile, other researchers conducted a study to determine the effect of used coffee grounds filler (CG 5%, 7.5% and 10%) on the physical and mechanical properties of a poly(lactic acid) (PLA) biocomposite film. The biocomposite film was fabricated by a twin-screw and blow-film extruder. Mechanical tests showed that the PLA/CG biocomposite film had increased elongation at break. This was observed while the hardness and brittleness of PLA decreased. The melt flow index (MFI) of the biocomposite film increased when the concentration of CG was higher [24].

In turn, the most popular areas of wood management are: construction material (panels, floors, roofs, doors, furniture), fuel, building material for ships, vehicles, pallets, everyday items (kitchen utensils, decorative figurines, toys), and paper industry (newspapers, books, paper packages) [25]. The main building blocks of wood and coffee are the same compounds, that is, hemicellulose, cellulose, and lignin, which mainly contain two or more hydroxyl groups in the molecule [26]. Wood processing produces many waste products, each of which has different properties due to the size. The main goal of mechanical processing is to obtain a suitable material for further production, that is, to preserve as many characteristics of wood as possible, which is most often related to the geometry of the particles [27]. The two by-products of wood processing are dust and wood particles [28], which are sawdust, wood chips, and sawmill chips. Due to the dynamic development of technology and the expansion of the scale of waste production, new ideas are created for the management of sawdust. Changes are also taking place due to increased awareness of environmental protection and the resulting threats. A very successful application of sawdust is the production of pellets. Wood pellets are a form of fuel for smaller-calibre energy installations. However, it is also used as animal feed, animal litter, fertilizers for gardens, and as coal fuel [29].

A relatively new solution is the use of sawdust in the plastics industry. Mirski et al. [28] used a solution based on the use of waste wood particles from primary wood processing as filler for polyurethane foam with an open cell structure. The applied filler was sawdust with a size fraction of 0.315–1.25 mm. This waste was added to the foam in an amount of 5, 10, 15 and 20 wt.%. The addition of sawdust to the open-cell polyurethane foam influenced the kinetics of the foaming process of the product. It was observed that the filler accelerates the onset of foam rise. In another case, Agnantopoulou et al. [30] prepared biodegradable composites from wood flour, sawmill residues and thermoplastic starch (Mater-BiTM and glycerol). Flour from four types of wood (spruce, pine, beech, and poplar), with three particle sizes, was used to produce the composites. Composites were characterized by mechanical properties measurement, scanning electron microscopy, water adsorption studies, thermal stability, and biodegradation. The addition of wood flour increased the tensile strength, modulus of elasticity, elongation, and thermal stability. On the other hand, the water absorption and biodegradation rates of the composites decreased. Almost all of the properties of the composites improved as the wood flour content increased and particle size decreased. Soft wood species (spruce, pine) are characterized by better mechanical and thermal properties and water adsorption, but with a lower biodegradation rate than hard wood species (beech, poplar) [30].

The growing awareness of environmental issues and strict legislation encourage the disposal of waste in an environmentally friendly manner, instead of traditional landfilling or incineration. Innovative and environmentally friendly methodologies for the exploitation of waste streams have attracted the attention of the scientific community. In a circular economy, waste is seen as a source of fine chemical recovery and the production of valuable metabolites through chemical and biotechnological processes. In the coming years, there is an urgent need to balance the industrial use of coffee grounds and sawdust. The motivation to conduct the research was the environmental aspects related to the use of waste materials of natural origin. Therefore, the main goal was to use coffee grounds and oak sawdust as innovative fillers for rigid polyurethane foams. In the case of polyurethane flooding foam, it is an innovative research direction. This idea has many benefits for the environment, scientists, and entrepreneurs in terms of ecology and economy. It gives the possibility of obtaining multifunctional ecological polyurethane composites. It is an innovative use of these fillers for rigid polyurethane flooding foams, which can be used, among others, in the construction, refrigeration, furniture, automotive, shipbuilding, heating, beekeeping, and decorative industries.

## 2. Materials and Methods

### 2.1. Materials

The characteristics of the substrates used to obtain multifunctional polyurethane composites (rigid polyurethane foam + filler) are shown in Table 1.

### 2.2. Preparation of Biofillers

An important stage of experimental research was the treatment of bioorganic materials/biofillers to prepare them for use as polyurethane foam fillers. 

The oak sawdust used came from a sawmill (Poland, town: Swarzędz), while the coffee grounds (*Coffea arabica*) came from a Starbucks coffee shop (Poland, Poznań). These materials were stored in a laboratory under appropriate conditions in a dry place. Initially, the materials were washed several times with distilled water. Then, they were dried at 105 °C for 24 h. A dryer UN55 (Memmert, Schwabach, Germany) was used for this process. In the last stage, the fillers were classified, that is, appropriate grinding and screening of the material through a sieve with a mesh diameter of 80 μm. Such materials were subjected to further studies. The X-ray scattering (XRD) method, using CuKα = 1.5418 Å at 30 kV and 20 mA anode excitation, was used to determine the supermolecular structure of the biofillers. XRD patterns were recorded for an angle range from 5 to 60 ° in the step of 0.04°/3 s (SmartLab SE diffractometer, Rigaku, Tokyo, Japan).

### 2.3. Preparation of Polyurethane Composite

To determine the physicochemical and functional properties, composites were obtained using two methods: free growth and mould shaping (imitation of an industrial process). In the free growth approach, polyurethane foams were synthesized in cups using a one-stage method. The reference sample was prepared as follows: 30 g of polyol and 36 g of isocyanate (isocyanate index for this group of foams, I_NCO_ = 1.08) were weighed and mixed with a mechanical stirrer for 10 s at 3600 rpm. The composites were prepared analogously: the process started by weighing the polyol and adding the appropriate amount of filler (2.5, 5, 10, 15, and 20 wt.%). The appropriate amount of isocyanate was added to the previously prepared mixture, and mixing was started. During the free rise of the polyurethane foam, the processing parameters were determined, that is, processing times, temperature as a function of time during foam expansion, and free density.

**Figure 1:**
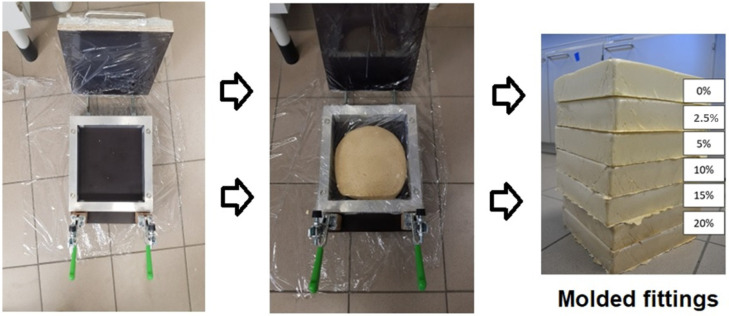
Preparation of moulded samples.

Polyurethane composites were also produced in the form of mouldings. The aim of the synthesis was to obtain foams with a density of 45 kg/m^3^, as this value is adequate for products used in industry. Previously prepared mould was heated to 50 °C. A reference sample was made with 70 g of component A and 84 g of component B (the amount of raw materials determined by calculations to obtain the density of the moulded part of 45 kg/m^3^). Composites were prepared similarly, using the same amount of components A and B, but in this case, filler (coffee grounds or sawdust: 2.5–20%) was added. The resulting mixture was poured into a rectangular laboratory mould with dimensions of 25 × 25 × 5 cm (3125 cm^3^) and closed. After 15 min, the mould was deformed. The scheme for obtaining the fittings is shown in Figure 1.

### 2.4. Characterization of a Polyurethane Composite

The tests carried out for both types of samples are given in Table 2. Additionally, the composites morphology of the obtained was determined. Scanning electron microscopy (SEM) images were taken to specify the structure of the particles and to reconstruct the morphology and microstructure of the tested materials. The Tescan MIRA3 scanning electron microscope (Princeton Gamma-Tech, Princeton, NJ, USA) was used as part of the research. An accelerating voltage of 12 kV was applied. To reduce sample charging, an approx. 20 nm carbon coating was deposited on them using a JEE 4B vacuum evaporator (Jeol, Peabody, MA, USA). Fourier transform infrared (FTIR) spectroscopy analysis was performed using a Vertex 70 apparatus (Bruker, Karlsruhe, Germany) to determine the presence of characteristic functional groups in the obtained materials.

All of the properties and parameters described in this study are intended to determine the potential for the application of innovative polyurethane materials (Table 2).

## 3. Results

### 3.1. Processing Times for Composites

**Figure 2:**
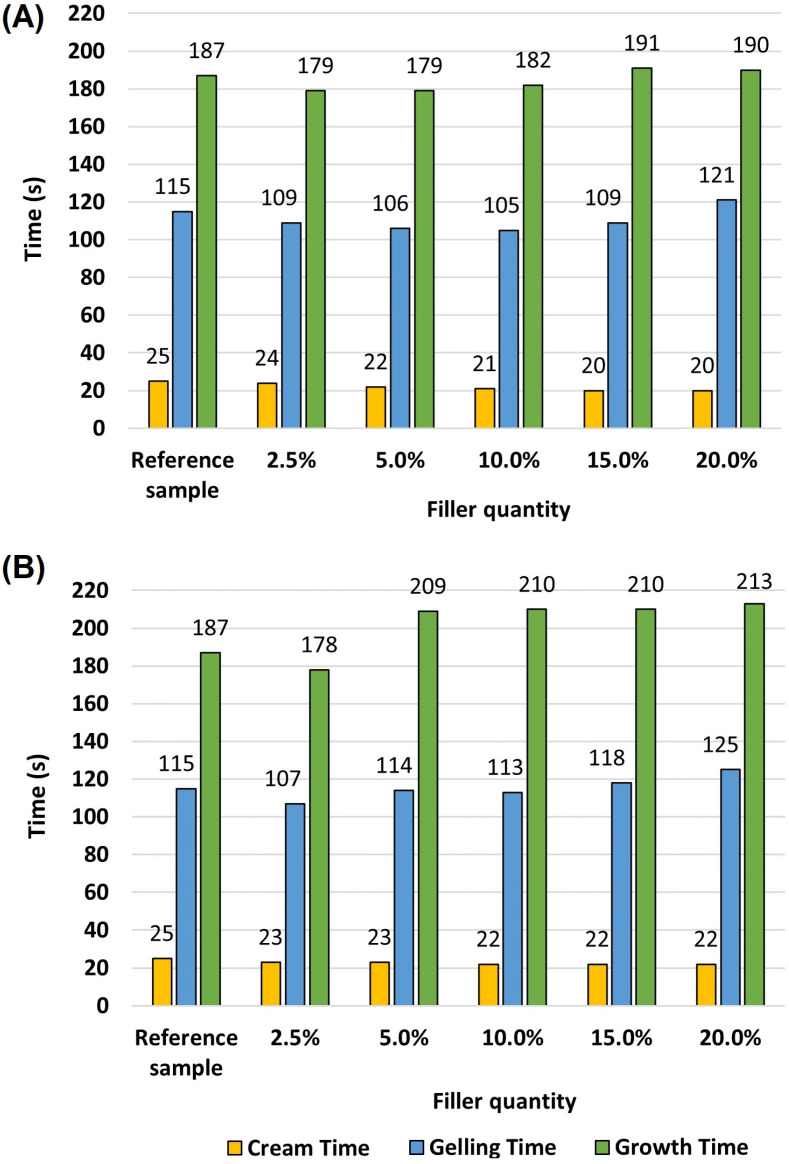
Characteristic processing times for polyurethane + coffee grounds (**A**) and oak sawdust (**B**).

During the preparation of polyurethane foam composites, in order to determine the effect of the bioorganic fillers used, the so-called processing times were measured (Figure 2). These times are extremely important in terms of the processing aspects of polyurethane foams.

It can be seen that the addition of filler affects the characteristic times of the foaming process. Incorporation of both coffee grounds and oak sawdust (at all loadings) accelerates the start of foam growth (cream time). This indicates that the filler increases the initial reactivity of the polyurethane system. The addition of filler in the range of 2.5 to 10% by weight influenced the time required for the system to reach the gelation stage. With the further increase in the content of coffee grounds and sawdust, this stage began to extend (gelling time). The greatest changes in gelation time were observed for samples with the highest amount of filler (20 wt.%). When sawdust (starting from 5 wt.%) was introduced to the foam, the end of foam growth took place later. In the case of coffee grounds, the differences were negligible. An important issue when considering the results of characteristic processing times is the amount of filler used. The analysis of the results indicates that the addition of sawdust did not significantly affect the start times of the obtained composites. On the other hand, longer gelation and end-of-growth times are probably due to the reduced expansion of the pores formed during the foaming process. The reason for this phenomenon is an increase in the viscosity of the mixture (polyol masterbatch with the addition of sawdust). Another cause is the formation of additional nucleation centres that, during the reaction, formed more bubbles. 

There are reports on rigid polyurethane foams with the addition of nut shells that showed similar relationships. The introduction of natural filler resulted in the shortening of the start time (from 45 to 40 s), but the gelation time and the end of growth were extended. The researchers speculated that it is due to an increase in the viscosity of polyol masterbatches [36].

Furthermore, nutmeg, a natural filler, was used for the production of polyurethane composite foams that showed antibacterial and anti-ageing properties [37]. In this case, completely different relationships were observed. The authors used a natural filler in the amount of 1, 2 and 5% by weight. All polyurethane composites were characterized by an extended time of foam start, gelation, and end of foam growth. For example, compared to the reference foam, the incorporation of 5 wt.% nutmeg increased the creaming time from 40 to 61 s and the end of expansion time from 230 to 249 s. The authors concluded that during the foaming process, solid nutmeg particles act as nucleation centres, resulting in the formation of a larger amount of bubbles. The higher viscosity of polymer systems containing natural filler limits the expansion of polyurethane composites. It results in an increase in free density (which will be further discussed in the following sections), interferes with the foaming process, and slows the rate of polymerization. This is in line with the findings of this study. 

### 3.2. Temperature as a Function of Time during Foam Expansion

**Figure 3:**
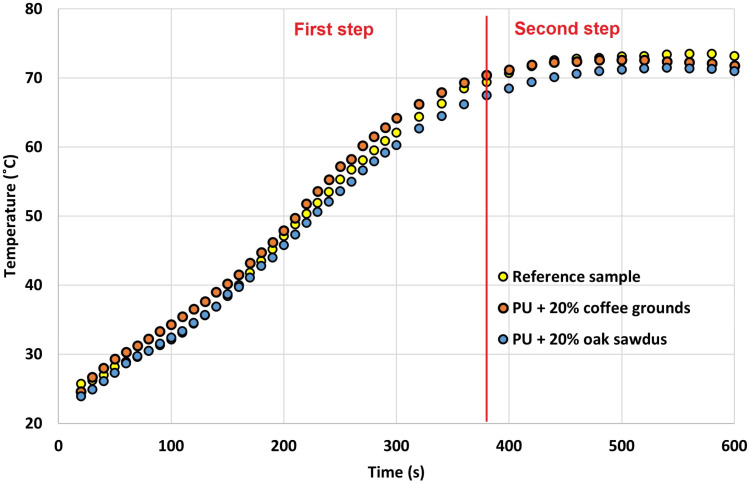
Temperature as a function of time during foam expansion of the reference sample and polyurethane composites.

It is well known that the synthesis of polyurethane materials is a highly exothermic reaction. Analysis of the temperature aspects of the foaming process is helpful in designing the optimal composition of the reaction mixture, thus avoiding overheating of the polyurethane foam core. Figure 3 shows the results of the temperature test during the synthesis of the reference sample and polyurethane foam with 20 wt.% of coffee grounds and oak sawdust.

When analysing the obtained graphs, two stages of polyurethane foam reactivity can be noted (this is marked in Figure 3). The first one concerns the intensive expansion of the foam and lasts up to about 380 s for all analysed samples. In the first stage, the polyurethane foam with additional coffee ground has a higher core temperature than the reference sample (the temperature is approximately 5 °C higher). On the other hand, in the case of the composite with the addition of oak sawdust, a lower temperature is observed (by approximately 4 °C compared to the reference sample). Most likely, the decrease in the temperature of the mixture containing sawdust results from the extension of the characteristic processing times, gel time and dry face for the addition of 20% filler (Figure 2B, notable extensions of these steps relative to the reference sample). Another aspect could be the presence of larger cells that enhanced air permeability and thus increased heat transfer in the foam. In the next stage of foaming, the process stabilizes (the second stage, after 500 s). The temperature equalization of all analysed composites and the reference sample are noticeable. None of the samples exceed a temperature above 100 °C. This causes an unfavourable process of polyurethane foam post-ignition. This is crucial when casting larger sizes (larger shapes) of the final product (lagging, blocks, or insulation boards).

Similar results were obtained by the group that conducted research on the reactivity of wood-reinforced polyurethane foams [28]. They noted a temperature drop during polymer synthesis from 95 to 82 °C. This resulted from a reduced amount of heat generated during the latent and growth phases, as well as from stabilization and maturation of the foam. The organic filler introduced into the polyurethane foam absorbed part of the heat generated, lowering the foaming temperature. Moreover, the presence of wood particles and their relatively large size undoubtedly limited both the growth of foam cells and their susceptibility to the foaming process.

### 3.3. Free Density of Composites

**Figure 4:**
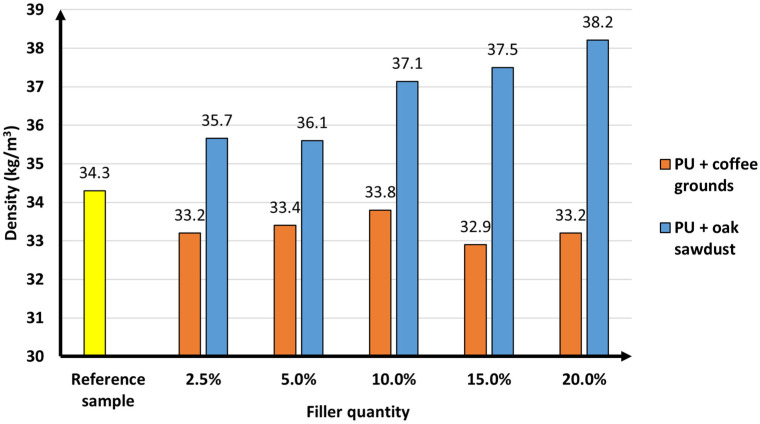
Free density of standard sample and polyurethane composites (±0.5 kg/m^3^).

Free density is the parameter that plays an important role in rigid polyurethane foams. It is highly responsible for the structure and functional properties of composites. Figure 4 presents the effect of bioorganic fillers on the free density of the polyurethane foams obtained.

The free density of the reference sample was 34.3 kg/m^3^. The free density of rigid foams with the addition of coffee grounds ranged from 33.2 to 33.8 kg/m^3^, while for sawdust filled samples, it ranged from 35.6 to 38.2 kg/m^3^.

Both fillers used affected the free density parameter differently. The addition of coffee grounds did not cause any significant changes, probably due to no noticeable change in viscosity of the composite. This was also confirmed by analysis of specific processing times. However, the use of a mixture of polyurethanes with oak sawdust reduced the expansion of the polyurethane mixture and, as a result, caused an increase in the free density of the polyurethane foams, increasing the viscosity of the mixture more than the coffee grounds. It can be concluded that the viscosity of the polyurethane mixture correlates with the formation of filler agglomerates in the cellular structure of the foam. This was discussed during the SEM photo analysis.

An analogous result was presented by Miedzińska et al. [38] who prepared polyurethane composites with ground plum seeds. The free density value for the reference foam was 38.2 kg/m^3^, and it was 43.5 kg/m^3^ for the maximum filler loading (5 wt.%). According to the authors, the addition of filler caused an increase in the dynamic viscosity of the polyurethane mixture, which impeded the expansion of bubbles and the formation of smaller-structured cells. Silva et al. [39] obtained polyurethane composites with cellulose fibres. It also resulted in an increase in the free density of the foams. The density of the reference sample was 29 kg/m^3^, while for polyurethane foam with the highest amount of cellulose fibres (16%), it was 37 kg/m^3^. The raw material used could cause a decrease in the reactivity of the system components, which affected the expansion of the foam, causing an increase in the density of the materials. In turn, Gawryla et al. [40] used casein (the main protein of cow’s milk) as a filler for cellular materials with properties similar to polyurethanes. The addition of this material (5–15 wt.%) significantly affected the density of the final product. The maximum amount of casein (15 wt.%) caused an increase in density by 96 kg/m^3^ compared to the standard sample. This was explained by the addition of a solid to the polymer matrices.

### 3.4. Compressive Strength of Composites

**Figure 5:**
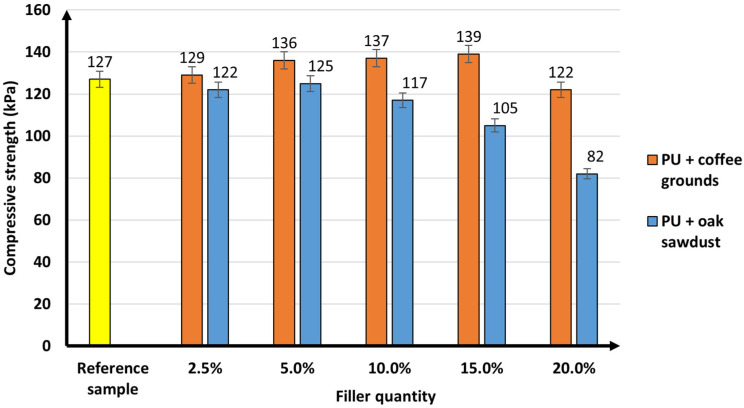
Compressive strength at 10% relative deformation of the reference sample and polyurethane composites.

The results of compressive strength measurements are closely related to the apparent density of the polyurethane foams. The compressive strength (at 10% shortening) of the composites was measured in the direction perpendicular to the growth of the foam. This is a crucial parameter that determines the mechanical and functional properties of foams. Figure 5 shows the results of this test.

The compressive strength for the reference sample was 127 kPa. For most samples, the introduction of coffee grounds into polyurethane foams resulted in an increase in compressive strength. The value of this parameter increased with the amount of CG added. However, at the highest loading of coffee grounds (20%), a significant decrease (to 122 kPa) was observed. The addition of small amounts of sawdust (up to 5%) resulted in a slight decrease in compressive stress (the results are comparable to those of the reference sample). However, as the filler content increased, the value of the discussed parameter dropped (to only 82 kPa for 20 wt.% of sawdust).

**Figure 6:**
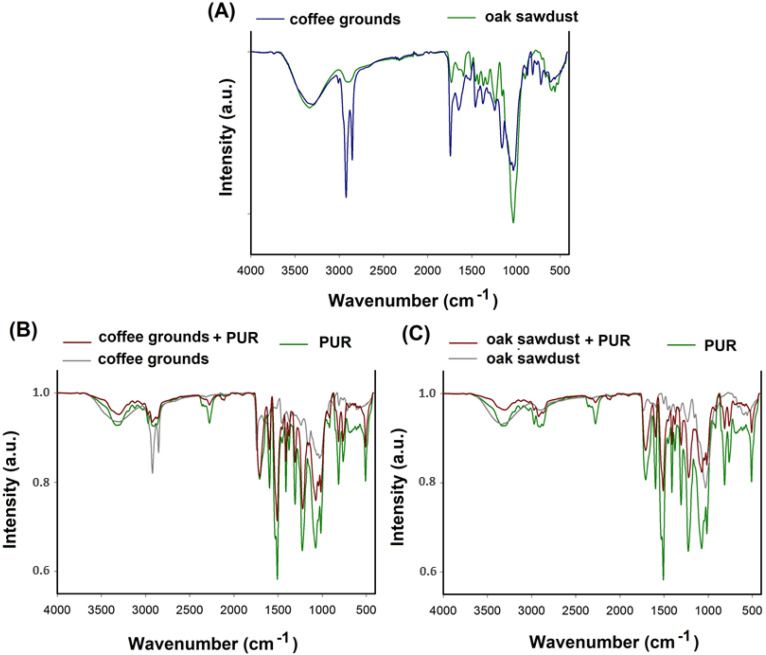
FT-IR spectra of powdered fillers (**A**), composites with the addition of coffee grounds (**B**), and oak sawdust (**C**).

Comparison of the compressive strength of materials prepared with coffee grounds and sawdust showed different relationships. The addition of coffee grounds caused an increase in compressive strength, and in the case of sawdust, a decrease. Such differences show that the mechanical properties are influenced not only by the apparent density of the foam but also by its structure. The decrease in compressive strength for sawdust samples may be due to the greater tendency for the particles to agglomerate. This leads to interfacial separation of the foam structure and promotes destruction of the cellular structure under compressive load. Coffee ground particles are less prone to agglomeration. The reinforcing effect can also be the result of interfacial adhesion between the coffee ground particles located at the edge of the cells and the polyurethane matrix, which facilitates stress transfer. The composition of the fillers used is of key importance in the interpretation. The main building blocks of wood are hemicellulose, cellulose and lignin, which contain -OH groups capable of reacting with isocyanates. This can cause defects in the foam structure. On the other hand, coffee grounds contain proteins, lipids, and, in smaller amounts than sawdust, lignin, cellulose, and hemicellulose. The polyurethane with coffee grounds was also more homogeneous (as proved by SEM photos, shown in the next section of this manuscript). In the case of using coffee grounds at 20 wt.% and oak sawdust at 15 and 20 wt.%, a change in foam hardness was noted. The resulting composites were softer. This most likely translates into a slight change in the cyanate index (input value for reference sample I_NCO_ = 1.08). The introduction of the filler slightly increased the number of -OH groups (from lignin and cellulose) that can react with the isocyanate. However, the reactivity and mobility of the hydroxyl groups present in the solid are limited. Bernardini [41] mentions in his research that lignin has limited reactivity of -OH groups due to steric impediments in the biopolymer structure, which prevented complete reaction with the isocyanate groups. This is mainly due to the structure of the position of the aromatic rings. Obtaining polyurethane foam with the addition of coffee grounds and oak sawdust is a very complex process. In order to improve the analysis, FTIR spectra of the fillers, the reference sample and the obtained composites were performed (Figure 6). 

**Figure 7:**
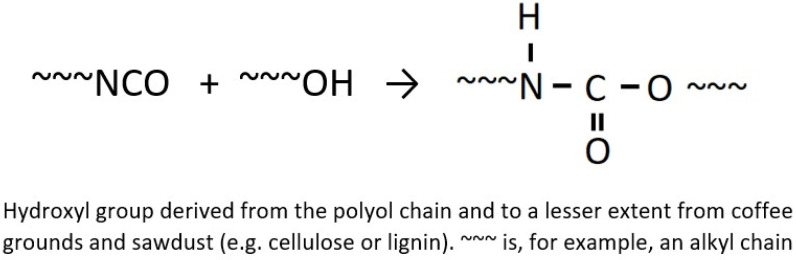
An example of a reaction occurring during the preparation of polyurethane composites.

The fillers used belong to the group of lignocellulosic materials, which have a complex and diverse structure. Analysing the spectra of the fillers (Figure A), it is possible to note the occurrence of wide bands of low intensity in the range of 3500–3000 cm^−1^ originating from stretching vibrations. This is due to the presence of O-H and N-H bonds present in lignin and cellulose. Absorption from 1200 to 900 cm^−1^ is associated with stretching vibrations of C-O and C-O-C bonds from hydrocarbons, e.g., cellulose and lignin. In their research, Olcay and Kocak [42] confirmed the presence of certain bonds assigned to cellulose. The researchers focused on the use of waste from rice cultivation as a filler for sound-absorbing foams. The reactivity and mobility of the hydroxyl groups present in the solid are limited. Jacopo Bernardini [41] mentions in his research that lignin has limited reactivity of -OH groups due to steric impediments in the biopolymer structure, which prevented a complete reaction with isocyanate groups. This is mainly due to the structure of the position of the aromatic rings. Analysing the FT-IR spectra for the reference foam, a characteristic band was observed in the region of 3500–3000 cm^−1^, which indicates the presence of vibrations of O-H stretching bonds in unreacted polyol and filler. In addition, the residues of polyols, specifically polyethers, can be confirmed by the presence of bands in the wavenumber range of 1270 and 1100 cm^−1^ assigned to the O-CO and C-O bonds. Such conclusions were also suggested in the research by Rivera-Armenta [43], who used various cellulose derivatives to obtain polyurethane foams. Then, vibrations of the N-H bond coming from the urethane moiety can also be observed. The band originating from N-H deformation vibrations is located at a wavelength of about 1510 cm^−1^. At a wavelength of about 2270 cm^−1^, a characteristic band appears, originating from the stretching vibrations of the isocyanate group -N=C=O. This proves the incomplete reaction of these groups. The band at the wavenumber of about 1700 cm^−1^ originates from the stretching vibrations, which indicate the presence of the C=O bond in the urethane groups. The FT-IR analysis of three composite samples and the standard sample showed similarity in the occurrence of characteristic functional groups, typical for the obtained polyurethane materials. Only a change in the intensity of the bands can be noted after the addition of waste fillers. In particular, one should focus on changing the intensity of the band responsible for the stretching vibrations of the -NCO group. This may suggest to some extent their reaction with hydroxyl groups derived from fillers. According to the FTIR analysis, the basic reaction that occurs during the preparation of composites can be presented below (Figure 7).

Uram et al. [44] synthesized rigid polyurethane foam with cellulose filler. They showed that the use of 1–3 php of cellulose had a negative effect on the mechanical properties of the material. The compression strength in the direction perpendicular to the growth of the foam was lower than for the reference sample. Pine sawdust (WS) was used as an additive in different papers [45]. The highest compressive strength value was observed for polyurethane foams with 40% WS. The parameter dropped as the amount of compressive strength increased for 85% of the filler, which was only 141 kPa. Sawdust was found to cause changes in the cellular structure of the foams. The filler tended to agglomerate, and cells with a smaller diameter were formed. This resulted in a decrease in compressive strength.

### 3.5. Water Absorption by Complete Immersion

**Figure 8:**
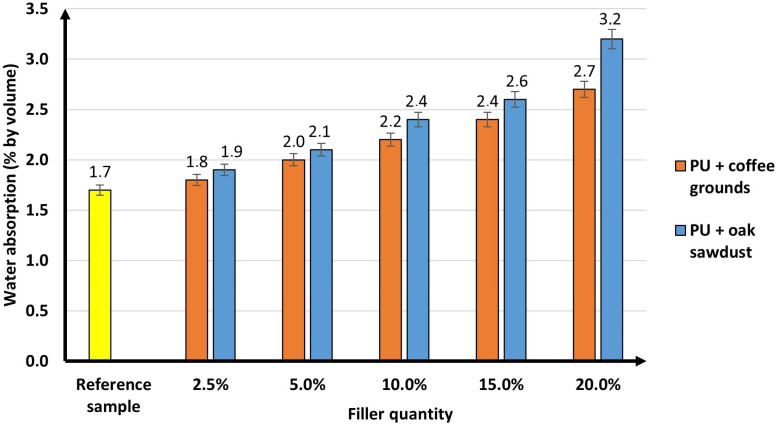
Water absorption by complete immersion for 24 h of the reference sample and polyurethane composites.

From the point of view of application, water absorption is one of the most important properties of polyurethane foams because the foam used in commercial applications cannot absorb water. Water absorption depends on the morphology of the foams and the hydrophobic nature of the filler. The tendency to absorb water may significantly affect its insulating and mechanical properties of the resulting composite. The results of the water absorption test for the reference sample and for the samples with the addition of bioorganic fillers are shown in Figure 8.

**Figure 9:**
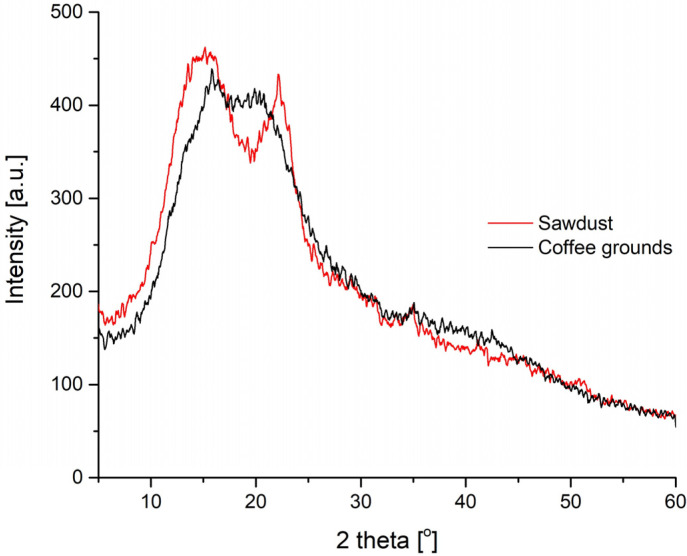
The XRD patterns recorded for sawdust and coffee grounds.

When analysing the obtained results, the fillers used have an analogous effect on the water absorption of the resulting polyurethane foams. In this case, an increasing trend was observed with the amount of filler added. However, for the obtained composites, the increase in the water absorption value was not significant. This may be due to disturbances in the cellular structure of the polyurethane materials caused by the addition of coffee grounds and sawdust. The water absorption values for sawdust are slightly higher than for coffee grounds. This can be caused by agglomerates of sawdust particles formed in the structure of the composites (SEM photos, Figure 9). The hydrophilic nature of the fillers used also had a significant impact on the results obtained. This applies in particular to oak sawdust, which in its structure, contains a significant amount of cellulose (much more than coffee grounds), showing hygroscopic properties. This is confirmed by the discussion of the results of the XRD analysis of the fillers. The XRD patterns recorded for sawdust and coffee grounds presented in Figure 8 show some important differences.

Pattern of sawdust shows two sharp peaks at 2Ө around 15° and 22°. There is also a bit overlapping peak present at 2Ө ≈ 16.5°. These peaks originate from crystallographic lattices of cellulose I [46]. In a sample of coffee grounds, the mentioned peaks are definitely shifted and are more indistinct. However, they are still characteristic for cellulose I, as also observed by Farhid et al. [47]. The crystallinity degree of coffee grounds was lower than for sawdust biofiller—23% versus 51 %. According to Dávila-Guzmán et al. [48], spent coffee grounds contain 34.6% of cellulose and 9.1% of lignin. Material tested by Caetano et al. [49] showed a bit different composition. The sample consisted of 33.6% cellulose and 13.8% lignin. In sawdust, the content of lignin and cellulose varies (depending on the wood type) in the range of 25.6–35.6% and 34.0–41.6%, respectively [50,51,52]. Overall, the XRD test suggests that coffee grounds had a lower content of crystalline cellulose content and a higher amount of amorphous substances, including lignin and hemicellulose. 

The foams used in industry must not exceed 3% of the water absorption value. The obtained composites were characterized by other significant functional properties, increasing the spectrum of application. Członka et al. obtained similar results [53] for polyurethane foam synthesis using eucalyptus fibres. They noted that the increase in water absorption may be related to the fact that natural fillers, especially sawdust, tend to agglomerate. For this reason, their structure may contain more voids, called “corridors”, through which water can diffuse inside the foam. 

### 3.6. Thermal Conductivity of Composites

Thermal conductivity is a key parameter determining property for polyurethane foam insulation. The higher the value of the thermal conductivity coefficient (λ), the worse the insulation parameters. The values of the thermal conductivity coefficient (λ) calculated for the reference sample and the composites are presented in Table 3. The thermal conductivity analysis was carried out at 10, 30 and 50 °C.

Given the data shown above, two trends are observed: the thermal conductivity slightly increases as more filler is added, and the measurement temperature increases, regardless of which filler was used. The addition of coffee grounds and sawdust caused a slight increase in λ for each temperature analysed. For foam with the maximum amount of CG additive (20%), the λ coefficient was higher by 7%, while for sawdust, it was 5% (when compared to the reference foam). The use of coffee grounds and sawdust resulted in a slight deterioration in the insulation properties of the biocomposites. Higher values of λ may be a consequence of the reduced content of closed cells, which facilitates thermal diffusion. This may be related to the agglomeration of filler particles and the change in the structure of the obtained composites. For rigid foams, this parameter should be in the range of 0.022–0.026 W/m·K (for the test temperature of 10 °C). Even though there is a slight increase, the composites produced are still within the given range. This is valuable information related to the application. Very often, the addition of fillers worsens the insulating properties of the polyurethane foams, e.g., pinewood bark [45] or nonfunctionalised lavender [54]. In this work, the introduction of fillers, even with a relatively high content (20%), resulted in the maintenance of excellent insulating properties, which will consequently determine the application potential of these innovative composite systems.

Similar findings were presented by Członka et al. [55] who tested the influence of walnut shells (WS) on the value of the thermal conductivity coefficient of rigid polyurethane foams. λ of polyurethane foams modified with WS ranged from 0.025 to 0.028 W/m·K. The addition of 1% walnut increased this parameter by approximately 4%, while for foams with 2 and 5 wt.% filler, it was 8% and 12%, respectively. The authors claimed that these differences were mainly related to changes in porosity and cell size.

### 3.7. Evaluation of Composite Flammability

**Figure 10:**
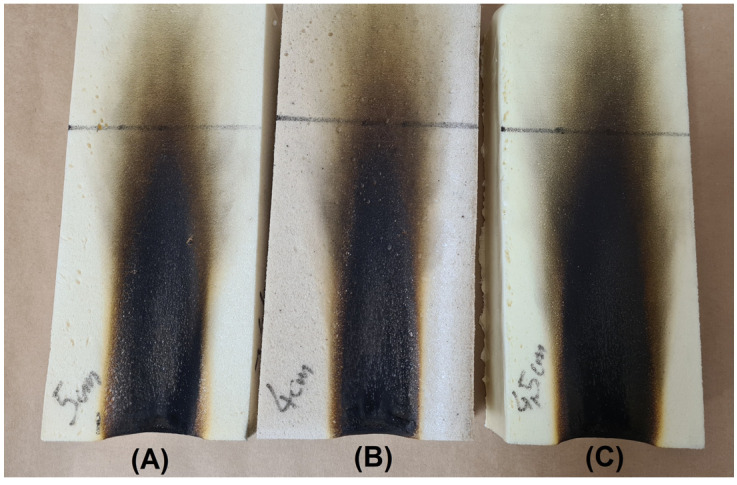
Photos of samples after the flammability test: reference sample (**A**), foam + coffee grounds 10 wt.% (**B**), and foam + sawdust 10 wt.% (**C**).

The biocomposites prepared in this study may be used as a pipe covering material and for the production of insulation boards used in the construction industry. In such an application, fire classification is crucial. Therefore, flammability tests were performed according to the PN EN 13501–1 standard. Table 4 and Figure 10 show the results obtained for reference foam and composites with coffee grounds and oak sawdust.

After being removed from the burner source (15 s), the samples stopped burning after a very short time, 17–18 s. The flame decay times of the composites are similar to those of the reference sample (18 s). In addition, Ranaweera et al. [56], who prepared rigid polyurethane foams with biopolyol, synthesized using 1-thioglycerol and orange peel extract, reported that the flame decay time in the obtained polyurethane foams was 19 s. 

A similar relationship can be observed when the height of the flame is discussed. This important parameter determines the spread of the flame to the higher parts of the composite, and for all samples, it was 17 cm. The last parameter examined was the ash width, which was slightly reduced and, compared to the reference sample, is a very satisfactory result. In summary, the addition of coffee grounds and oak sawdust not only did not cause significant differences in flammability, but it also did not deteriorate the flammability of the polyurethane foams. This may be a result of the chemical structure and closed cell structure of the peptides present in the applied fillers. Materials can be characterized by flammability class E, and this is crucial in terms of the application of the obtained composites. 

### 3.8. Evaluation of Composite Morphology

**Figure 11:**
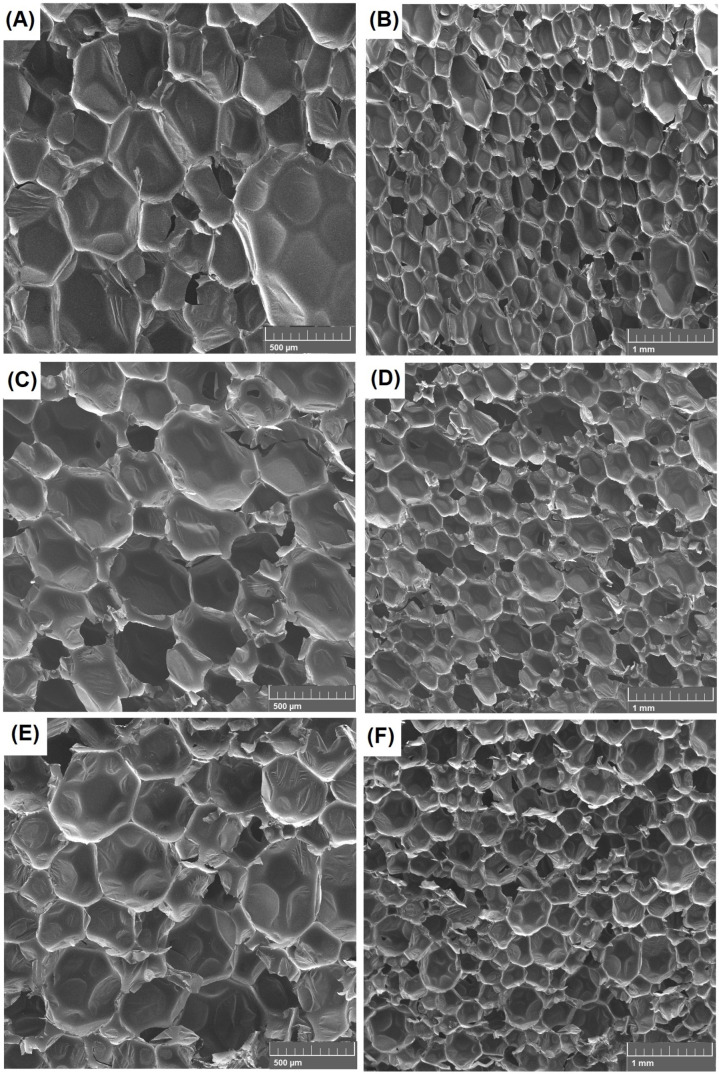
SEM photos: reference sample (**A**,**B**), foam + coffee grounds 10 wt.% (**C**,**D**) and foam + sawdust 10 wt.% (**E**,**F**) on a scale 500 µm and 1 mm.

SEM photos (Figure 11) were taken to determine the relationship between the structure of the composites and the mechanical, thermal, and physical properties of the materials. 

The structure of the reference sample is almost homogeneous, with a high content of closed cells. The addition of 10% coffee grounds (Figure 11C,D) leads to the formation of a more homogeneous polyurethane structure than when the same amount of sawdust (Figure 11E,F) is used. Composites with sawdust had a slightly larger diameter of closed cells (Figure 11E,F). For sawdust-filled composites, the foam morphology became less homogeneous, and a greater number of damaged and small cells were present. In addition, it is possible to create larger clusters of sawdust. This filler contains a large amount of cellulose and lignin capable of forming hydrogen bonds. This increases the likelihood of aggregates that may affect the cellular structure of the foam. Solid particles can reduce the nucleation energy by changing the nucleation from homogeneous to heterogeneous. A lower nucleation barrier favours the formation of large numbers of smaller cells, which, in turn, tend to coalesce into larger ones. That might have been the reason for the deterioration of the compressive strength mentioned in previous chapters. In the case of coffee ground composites, a smaller amount of filler particles embedded in the matrix was observed (Figure 11C,D). It is possible that the interfacial interaction between the filler and the polyurethane matrix favours the formation of a more uniform structure, which in turn leads to the growth of cells and the formation of closed pores. It can be concluded that this is the reason behind the achievement of good mechanical and insulating properties. The difference in the structure of the obtained composites is related to the composition and chemical structure of the bioorganic fillers used. Coffee grounds contain more active and volatile compounds. The appropriate correlation between the filler concentration, the viscosity of the polyurethane system, and the distribution of natural additive particles in the polymer matrix appears to be the key factor in obtaining polyurethane materials with homogeneous morphology. 

The morphology of polyurethane foams with fillers was studied quite extensively. Leng et al. [57] performed research on polyurethane foam modified with cellulose nanofibres (CNF). Using a scanning electron microscope, the microstructures of the polyurethane and CNF-polyurethane samples were analysed. There was a noticeable trend showing that an increase in the amount of nanofibres resulted in a decrease in the average cell size of the tested samples. For the reference sample, this value was 741 µm, and for the sample with the highest filler content, it was 589 µm. The smaller size of the closed cells in the CNF-modified polyurethane foam indicated a larger number of cells in a given mass that were distributed more homogeneously and thus enhanced the mechanical strength. Composites were characterized not only by smaller cell sizes, but also by fewer defects. Członka et al. [58] worked on polyurethane foams with the addition of coir fibres. The effect of the filler on the cellular morphology of polyurethane composites was assessed. The structure of the reference foam was uniform with a large number of closed cells. After coconut fibre was added, the closed cell structure of the composites was still well preserved. Among all modified polymer composites, the polyurethane composite reinforced with 5 wt.% coconut fibre filler showed the highest number of damaged cells and the most heterogeneous cell size.

## 4. Conclusions

This study has indicated that waste materials of natural origin, such as coffee grounds and oak sawdust, can be used successfully for rigid polyurethane flood foams. The addition of fillers affected the stages of formation and moulding of polyurethane composites. There is a notable increase in the polymer formation time (cream time, gel time, glue drying time) when using sawdust. However, for the coffee grounds, the differences were not that significant. The innovative polyurethane foam with the addition of sawdust was characterized by an increased density compared to the reference sample, 34.3 kg/m^2^ (the highest being 38.2 kg/m^2^ at 20 wt.% sawdust). It can be seen that the free density value increased with the amount of added filler. The coffee grounds did not show an effect on the density when free standing. The results of the composite analysis showed a different dependence on the influence of the added fillers on the mechanical properties. Increasing the amount of added coffee grounds had a positive effect on compressive strength values. The best result was obtained with 15% mass of this filler (139 kPa). The filler in the form of sawdust decreased the compressive strength value of the composites obtained in relation to the reference foam. The results of the mechanical tests of polyurethane foams (2.5–15% of the filler) are acceptable for industrial use. The introduction of coffee grounds and sawdust to the polyurethane mixture caused a slight deterioration of the insulating properties of the composites. However, the values of thermal conductivity obtained in this study are within the industrial requirements of 0.022–0.026 W/m·K. This is an extremely important relationship due to the use of these materials for insulation purposes. The results of the flammability tests confirmed that the addition of bioorganic materials did not adversely affect the fire resistance of the foams. The results were analogous to those of the reference sample without the addition of fillers. The introduction of natural fillers (coffee grounds and oak sawdust) into the polymer matrix is an effective method of maintaining the physicochemical and functional properties (water absorption, insulation, and fire resistance) of rigid polyurethane pouring foams. The key was the addition of coffee grounds (up to 15 wt.%) where better mechanical properties and more precisely compressive strength were obtained compared to the reference sample. Polyurethane composites with up to 15 wt.% coffee grounds and up to 10 wt.% oak sawdust can be used successfully in industry. Composites modified with coffee grounds and sawdust are interesting from a technological, ecological, and economic point of view. This significantly increases the range of industrial applications of foams as effective insulation materials.

## Figures and Tables

**Table 1:** Components used and their physicochemical properties.

Component A	Component B	Fillers
POLYOL–commercial blend of short- and longchain polyols with additives	ISOCYANATE–polymeric 4,4-diphenylene methane diisocyanate (pMDI)	fillers of natural origin as industrial waste
• density 1.14 (g/cm^3^), • viscosity 500 (mPa·s), • polyol blend up to 65%, • flame retardant (tri (2-chloro-1-methylethyl) phosphate) up to 10%, • low boiling organic liquid (1,1,1,3,3-pentafluorobutane) up to 10%, • catalysts (N, N-dimethylcyclohyxylamine) up to 5%, • stabilizer up to 5%, • water up to 7%.	• NCO groups content approx. 32%, • density 1.23 (g/cm^3^), • viscosity 210 (mPa·s).	• coffee grounds (*Coffea arabica*), • oak sawdust (Quercus L.).

**Table 2:** Conducted analyses of polyurethane composites.

Type of Fitting (Composite)	Type of Analysis	According to the Norm
Free growth samples	Processing times: Start time—the time after which the mixture reaches the structure of a cream and the upward expansion of the foam is initiated; Gelation time—the time after which the viscosity of the mixture increases and internal crosslinking of the foam begins; Growth end time—the time after which the foam growth ends).	-
	2. Temperature as a function of time during foam expansion (rigid polyurethane foam was measured using a TES-1312A thermocouple with recorder).	-
	3. Density of the polyurethane foam with free growth.	PN-EN 1602 [31]
Moulded samples	4. Determination of compressive strength at 10% strain using the Allround-Line Z020 TEW Zwick and Roell.	PN-EN 826 [32]
	5. Determination of water absorption by complete immersion for 24 h.	PN-EN 12087 [33]
	6. Determination of thermal conductivity at 10, 30 and 50 °C using Taurus TCA 300, Netzsch Taurus Instruments.	PN-EN 12667 [34]
	7. Fire classification of construction products and building elements—Part 1: Classification based on reaction to fire tests.	PN-EN 13501–1:2019–02 [35]

**Table 3:** Results of the thermal conductivity coefficient (W/m·K) for the reference sample and composites with coffee grounds and oak sawdust.

	Amount of Filler (%)	Analysed Temperature (°C)		
		10	30	50
	Reference sample	0.02238	0.02562	0.02881
Coffee grounds	2.5	0.02249	0.02584	0.02899
	5.0	0.02287	0.02621	0.02937
	10.0	0.02319	0.02668	0.02968
	15.0	0.02341	0.02694	0.02999
	20.0	0.02391	0.02745	0.03065
Oak sawdust	2.5	0.02244	0.02578	0.02895
	5.0	0.02251	0.02593	0.02922
	10.0	0.02261	0.02618	0.02954
	15.0	0.02295	0.02658	0.02982
	20.0	0.02350	0.02697	0.03031

**Table 4:** Flammability test results.

Type of Analysis	Reference Sample	Foam + Coffee Grounds	Foam + Sawdust
Flame decay time (s)	18	17	18
Flame height (cm)	17.0	17.0	17.0
Ash width (cm)	5.0	4.0	4.5

## Data Availability

Data available on request.
